# Galectin-3 as a Prognostic Biomarker in Patients with First Acute Myocardial Infarction without Heart Failure

**DOI:** 10.3390/diagnostics13213348

**Published:** 2023-10-31

**Authors:** Rada M. Vucic, Olivera M. Andrejic, Dragana Stokanovic, Tatjana Jevtovic Stoimenov, Lana McClements, Valentina N. Nikolic, Miodrag Sreckovic, Mirjana Veselinovic, Srdjan Aleksandric, Viseslav Popadic, Marija Zdravkovic, Milan Pavlovic

**Affiliations:** 1 Department of Internal Medicine, Faculty of Medical Sciences, University of Kragujevac, Svetozara Markovica Street 69, 34000 Kragujevac, Serbia; sreckovic7@gmail.com (M.S.); veselinovic.m@sbb.rs (M.V.); 2 Clinic of Cardiology, University Clinical Centre Kragujevac, Zmaj Jovina Street 30, 34000 Kragujevac, Serbia; 3 Clinic for Pulmonology, University Clinical Centre Kragujevac, Zmaj Jovina Street 30, 34000 Kragujevac, Serbia; olivera.andrejic@gmail.com; 4 Department of Pharmacology and Toxicology, Medical Faculty, University of Nis, Bulevar dr Zorana Djindjica 81, 18000 Nis, Serbia; dstokanovic@gmail.com (D.S.); valentina@medfak.ni.ac.rs (V.N.N.); 5 Institute of Biochemistry, Medical Faculty, University of Nis, Bulevar dr Zorana Djindjica 81, 18000 Nis, Serbia; tjevtovic@yahoo.com; 6 School of Life Sciences, Faculty of Science, University of Technology Sydney, Sydney, NSW 2007, Australia; lana.mcclements@uts.edu.au; 7 Cardiology Clinic, University Clinical Center of Serbia, 11000 Belgrade, Serbia; srdjanaleksandric@gmail.com; 8 Faculty of Medicine, University of Belgrade, 11000 Belgrade, Serbia; sekcija.kardioloska@gmail.com; 9 Clinic for Internal Medicine, University Clinical Hospital Center Bezanijska Kosa, 11080 Belgrade, Serbia; viseslavpopadic@gmail.com; 10 Department of Internal Medicine—Cardiology, Medical Faculty, University of Nis, Bulevar dr Zorana Djindjica 81, 18000 Nis, Serbia; milan1.pavlovic1@gmail.com; 11 Clinic for Cardiovascular Diseases, University Clinical Centre Nis, Bulevar dr Zorana Djindjica 48, 18000 Nis, Serbia

**Keywords:** galectin-3, acute myocardial infarction, major adverse cardiovascular events

## Abstract

Background: Galectin-3 (Gal-3) is a biomarker involved in a wide range of diseases including cardiac remodeling following acute myocardial infarction (AMI). Identification of prognostic markers in patients with AMI can guide strategies towards improved survival and quality of life. Methods: Our study included 59 patients with AMI and a preserved ejection fraction. We determined the Gal-3 plasma concentration within 24 h of chest pain onset from the aortic root, femoral/radial artery, coronary sinus and cubital vein. Major adverse cardiovascular events (MACEs) were evaluated at six months follow-up. Results: MACE at six months post-AMI was recorded in 20 patients (34%). The Gal-3 plasma concentration from the aortic root and the femoral/radial artery were independent predictors of MACE at six months follow-up after the first AMI (OR 1.228; 95%CI: 1.011–1.491; *p* = 0.038; OR 3.438; 95%CI: 1.275–9.265; *p* = 0.015). ROC analysis identifies the Gal-3 plasma concentration from the aortic root as a better predictor of MACE or death (cut-off ≥ 10.86 ng/mL; AUC 0.858; 95%CI: 0.744–0.973; *p* < 0.001) than Gal-3 plasma concentration from the femoral/radial artery (cut-off ≥ 10.18 ng/mL; AUC 0.742; 95%CI: 0.596–0.888; *p* = 0.006). Conclusion: the Gal-3 plasma concentration in patients with AMI determined during coronary angiography, especially from the aortic root, within 24 h after chest pain onset is a valuable biomarker of prognosis at six months follow-up.

## 1. Introduction

Galectin-3 (Gal-3) is a member of the lectin family and is involved in a wide range of inflammatory, autoimmune diseases, acute cardiovascular events, atrial fibrillation (AF) and myocardial fibrosis [1,2]. Its important role in tissue repair following AMI has been demonstrated in animal models and in a small number of human samples [3]. Thus, Gal-3 is directly involved in adverse myocardial remodeling [4,5] and associated with major adverse cardiovascular events (MACEs) [6,7]. The crucial role of Gal-3 in myocardial fibrosis is linked to activated macrophages and damaged cardiomyocytes, which are the primary sources of Gal-3 [8]. Increased expression of Gal-3 is closely associated with the development and progression of cardiovascular diseases [9]. Conversely, in a study conducted in Poland that enrolled 107 clinically stable patients with dilated cardiomyopathy who were followed for approximately 4.8 years, the prognostic value of Gal-3 was not of significance [10]. However, this study excluded patients with coronary artery disease, valvular heart disease, connective tissue disease, significant renal insufficiency and inflammatory or infectious disorders. It has been found that higher concentrations of Gal-3 are related to higher mortality in the general population and HF patients [11,12,13].

In our previously published study, we showed that Gal-3 plasma concentration 24 h post-AMI was increased in those individuals who develop adverse myocardial remodeling six months post-AMI [13]. Adverse myocardial remodeling is followed by heart failure with a reduced left ventricle ejection fraction (HFrEF). Therefore, the aim of this study was to examine the prognostic value of Gal-3 from arterial and venous blood and central and peripheral blood in patients with AMI at six months follow-up.

## 2. Materials and Methods

### 2.1. Study Participants

This prospective study included 59 AMI patients (41 males (69.5%); mean age 65 ± 9 years; range: 45–81 years) scheduled for urgent coronary angiography. Patients were hospitalized in the University Clinical Centre of Nis and the University Clinical Centre of Kragujevac from December 2016 to November 2018 as a result of the first AMI with preserved left ventricle ejection fraction (LVEF > 50%). Patients were admitted within the first 24 h of chest pain onset. Diagnostic procedures (coronary angiography, electrocardiography, echocardiography) and myocardial revascularization by percutaneous coronary intervention (PCI) were performed in accordance with the institution’s guidelines and International Cardiology Association’s recommendations [14]. We excluded patients with any previous AMI; any previous PCI; previous aorto-coronary by-pass grafting surgery (CABG); congenital and acquired valvular heart disease; left ventricular ejection fraction (LVEF) <50%; left ventricular hypertrophy; cardiomyopathies (dilated, hypertrophic, restrictive); autoimmune diseases; impaired renal function (GFR < 30 mL/min); systemic inflammatory diseases; cancer; or if a participant refused to take part in the study. Written informed consent was obtained from all participants prior to their inclusion in this study. The research was performed in accordance with the Helsinki Declaration and approved by institutional ethics committees (Clinical Centre of Kragujevac and Clinical Centre of Nis).

### 2.2. Study Design

Blood sampling was carried out within 24 h of chest pain onset during coronary angiography and PCI in patients with or without ST-elevation myocardial infarction (STEMI and NSTEMI). Gal-3 plasma concentration was measured in the aortic root, femoral/radial artery and the right atrium near the coronary sinus and cubital vein on day one. Plasma was separated from the whole blood by centrifugation at 3000× *g* for 10 min at 25 °C, aliquoted and frozen at −80 °C. Commercially available ELISA kit (BGM, Inc., Waltham, MA, USA) was used to determine Gal-3 plasma concentration according to the manufacturer’s instruction.

The following parameters were evaluated using echocardiography (biplane area-length echocardiography method—Simpson’s formula) on day one: left ventricle end diastolic volume (LVEDV), left ventricle end systolic volume (LVESV), LVEF, diameter of left atrium (LA), mitral valve inflow (E/A) and tissue Doppler velocity (E/E’). Biomarker results were unavailable to physicians who performed echocardiographic assessments. Routine blood tests were performed within 24 h of chest pain onset. Biomarkers of myocardial necrosis, troponin and creatine kinase isoenzyme MB (CK-MB) were quantified only to confirm the diagnosis of AMI, without serial testing over time. Major adverse cardiovascular events (MACEs) are defined as a composite of death, re-AMI, cerebrovascular insult and re-hospitalization due to heart failure or malignant arrhythmias (ventricular tachycardia, ventricular fibrillation, atrio-ventricular block, asystole).

### 2.3. Statistical Analysis

The data were processed using the Statistical Package for Social Sciences (SPSS, v. 21.0; Chicago, IL, USA). Continuous variables were presented as the mean value ± standard deviation or median ± interquartile range and categorical variables as count with percentages. Continuous variables were compared with unpaired Student’s *t* test and or Mann–Whitney U-test, depending on the normality of continuous data distribution. Normal distribution of continuous variables was evaluated by both Kolmogorov–Smirnov and Levene tests. Categorical variables were compared with chi-squared or Fischer’s exact test, depending on group sizes. Pearson’s correlation coefficient was used for correlation of continuous variables and multivariable logistic regression analysis for the influence of putative risk variables on outcomes. We expressed these results as adjusted odds ratios with respective 95% confidence intervals. Receiver operating characteristic (ROC) curves with 95% confidence intervals (CI) were used to estimate diagnostic accuracy, while the highest Youden’s index (sensitivity + specificity − 1) was used to assess the best cut-off value of Gal-3 concentrations for discrimination of patients with and without MACE at six months follow-up. The statistical significance was determined as two-sided value of *p* < 0.05.

## 3. Results

### 3.1. Baseline Characteristics of This Study’s Patients and Major Adverse Cardiovascular Events (MACEs)

Baseline characteristics of this study’s patients are presented in Table 1. Thirty-eight patients (64%) had STEMI, whereas 21 patients (36%) had NSTEMI. The events of MACE including death were associated with higher Gal-3 concentrations in all four locations: aortic root (13.9 [10.8–16.2] vs. 8.2 [5.9–9.9] ng/mL, *p* < 0.001), femoral/radial artery (11.55 ± 3.15 vs. 8.53 ± 3.46 ng/mL, *p* = 0.005), coronary sinus (12.56 ± 4.40 vs. 8.43 ± 2.57 ng/mL, *p* = 0.001) and cubital vein (12.06 ± 3.80 vs. 8.05 ± 2.21 ng/mL, *p* < 0.001).

Patients experiencing MACE appeared to be older (69 ± 10 vs. 63 ± 8 years, *p* = 0.013) with a higher frequency of pre-existing AV block (17 vs. 0%, *p* = 0.025). On admission, they had both a lower systolic and diastolic blood pressure (115 ± 26 vs. 137 ± 27 mmHg, *p* = 0.005; 68 ± 15 vs. 79 ± 17 mmHg, *p* = 0.023, respectively), creatinine clearance (64.32 ± 29.95 vs. 91.79 ± 21.29 mL/min, *p* < 0.001), LDL (3.19 ± 1.33 vs. 3.86 ± 1.02 mmol/L, *p* = 0.042), red blood cell count (4.30 ± 0.58 vs. 4.73 ± 0.51 × 10^12^/L, *p* = 0.007) and hemoglobin (120.53 ± 17.91 vs. 144.44 ± 14.62 g/L. *p* < 0.001). They also had increased levels of urea (9.6 [7.4–13.3] vs. 5.8 [4.6–6.9] mmol/L, *p* = 0.003), creatinine (115.5 [79.8–167.8] vs. 86.0 [77.0–93.8] μmol/L, *p* = 044), NT-pro-BNP (2250.0 [275.2–6492.8] vs. 372.5 [180.2–1979.5] pg/mL, *p* < 0.01) and glycaemia (8.2 [5.0–12.6] vs. 6.0 [5.3–7.0], *p* = 0.040). In addition, patients who suffered MACE were more frequently on furosemide therapy (53 vs. 22%, *p* = 0.030) (Table 1).

The presence of MACE at six months post-AMI was recorded in 20 patients (34%) (Table 2).

Major adverse cardiovascular events (MACEs) are defined as a composite of death, re-AMI, cerebrovascular insult and re-hospitalization due to heart failure or malignant arrhythmias. Malignant arrhythmias are defined as ventricular tachycardia, ventricular fibrillation, atrio-ventricular block or asystole.

### 3.2. Prognostic Value of Galectin-3 Plasma Concentration Measured at Four Different Sites in AMI Patients

Multivariate logistic regression analyses (backward method) were performed for all significant univariate predictors and galectin-3 level measured in the aortic root, the femoral/radial artery, the right atrium near the coronary sinus and the cubital vein (Table 3). In this manner, the Gal-3 plasma concentrations measured in the aortic root and the femoral/radial artery were identified as independent predictors of MACE or death at six months follow-up after the first AMI (OR 1.228; 95%CI: 1.011–1.491; *p* = 0.038; OR 3.438; 95%CI: 1.275–9.265; *p* = 0.015). The Gal-3 plasma concentrations at all four sites were not considered in the same multivariate analysis due to high correlation and multi-collinearity (aortic root vs. coronary sinus: r = 0.685, *p* < 0.001; aortic root vs. cubital vein: r = 0.877, *p* < 0.001; coronary sinus vs. cubital vein: r = 0.883, *p* < 0.001, respectively).

**Figure 1:**
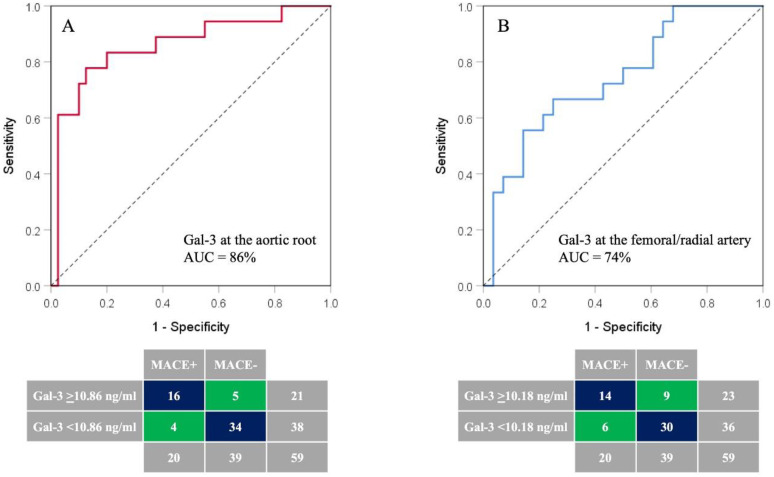
The ROC curve analysis of Gal-3 plasma concentrations measured in the aortic root (**A**) and the femoral/radial artery (**B**) in the identification of AMI patients with likelihood of 6-month occurrence of MACE or death. AUC—area under curve; Gal-3—galectin-3.

Based on ROC analyses (Figure 1), the Gal-3 plasma concentration measured in the aortic root was identified as a better predictor of MACE including death at six months post-AMI (AUC 0.858; 95%CI: 0.744–0.973; *p* < 0.001) than the Gal-3 plasma concentration measured in the femoral/radial artery (AUC 0.742; 95%CI: 0.596–0.888; *p* = 0.006).

The optimal cut-off value for the Gal-3 plasma concentration measured in the aortic root for detection of AMI patients with an increased risk of MACE or death at six months post-AMI was ≥10.86 ng/mL, with sensitivity, specificity, positive and negative predictive values of 80%, 87%, 76% and 89%, respectively (Table 4). The overall accuracy of this test was 85%. Similar results were obtained for the optimal cut-off value of Gal-3 plasma concentration measured in the femoral/radial artery ≥10.18 ng/mL but with lower sensitivity, specificity, positive and negative predictive values and overall accuracies of 70%, 77%, 61%, 83% and 75%, respectively (Table 4).

## 4. Discussion

Our study compared the prognostic value of Gal-3 plasma concentrations in arterial and venous blood and central and peripheral blood in patients with first AMI and preserved LVEF, in the first 24 h after chest pain onset. The main finding of this study is that the Gal-3 plasma concentrations measured at the aortic root within 24 h of chest pain onset in AMI patients are a valuable biomarker of prognosis at six months follow-up. The optimal cut-off value of ≥10.86 ng/mL for Gal-3 plasma concentration measured at this site has the best sensitivity and specificity for stratifying AMI patients with increased risk of MACE or death at six months post-AMI. If the Gal-3 plasma concentration from the aortic root is quantified during coronary angiography on the first day following AMI, we can stratify patients with an increased Gal-3 plasma concentration (≥10.86 ng/mL) who are very likely to develop MACE or die within the first six months following AMI.

The prognostic value of Gal-3 in peripheral blood for mortality and other adverse outcomes in patients with AMI has been reported in other trials [15,16,17,18,19,20,21,22,23]. Gagno G and colleagues included 469 patients with AMI (STEMI and NSTEMI) and collected venous blood samples within 8 h from admission. The primary outcomes were recurrent angina, re-MI and all-cause mortality within one year after the PCI. The Gal-3 was an independent predictor of one-year mortality in patients who were alive 30 days after the AMI (OR: 4.25; 95%CI: 2.12–8.5; *p* < 0.01). A multivariate model including age, LVEF, Gal-3 and renal function at discharge was comparable to the GRACE score for predicting one-year mortality and demonstrated a slight improvement with AUC 0.84 (95%CI: 0.78–0.90) over the GRACE score with AUC 0.82 (95%CI: 0.75–0.88) [15]. Furthermore, Asleh R and colleagues reported high prognostic value of Gal-3 during a mean follow-up of 5.4 years following AMI. Gal-3 was an independent predictor of mortality and HF post-MI. Elevated Gal-3 was associated with increased mortality with 5-year estimates of 10.2%, 24.4% and 51.9%, depending on Gal-3 concentration cut-off (*p* < 0.001). The association followed a dose–response pattern with a 30% increased risk of death for each 10-unit increase in Gal-3 (HR 1.30, 95%CI: 1.24–1.36) [16].

Other studies similar to ours have shown aligned results for Gal-3 with a cut-off value of 7.67 ng/mL and sensitivity and specificity of 74.5% and 72.4% (AUC 0.78), respectively, for predicting 30-day MACE after AMI [18]. Idzikowska K and colleagues followed up on 96 AMI patients for one year and reported nine MACEs (cardiac death, re-MI and need for unscheduled PCI). No significant differences in the concentration of Gal-3 on day one of hospitalization were found between patients who experienced late MACE and uneventful survivors (*p* = 0.56). However, the ROC curve analysis found that a Gal-3 concentration assessed on admission with a cut-off value of 23.183 ng/mL (95%CI: 2.664–54.059) was a strong predictor of MACE (AUC 0.75, *p* = 0.0061) and death (AUC 0.854, *p* < 0.001) within one year after discharge. ROC curve analysis revealed also that Gal-3 concentration collected on admission may also be used as a strong predictor of death (AUC = 0.854, *p* < 0.001) [21]. Tyminska A and colleagues included 117 STEMI patients treated with PCI. Gal-3 and sST2 were sampled 72 to 96 h after admission due to STEMI. The patients were followed up to the primary endpoint (cardiovascular death or heart failure hospitalization at 1 year). Both Gal-3 and sST2 were predictors of the primary endpoint and of both CV death and HF hospitalizations alone [22].

In our study, a lower hemoglobin level and reduced creatinine clearance were found to be independent predictors of MACE or death at six months follow-up after the first AMI. Kang et al. found that anemia was an independent predictor of MACE in patients with acute coronary syndrome (ACS) [23]. Higher creatinine serum concentration or reduced creatinine clearance are associated with increased risk of both short- and long-term MACE or death [24]. According to a study from the Canberra Hospital registry 2016, the mortality rate following AMI during the first year was 7.1%, whereas during the second year it was lower, 2.05%, but only 25% patients with fatal events had LVEF ≤ 35% [25]. In our study, no difference in LVEF was observed between the groups with and without MACE including death. Therefore, only some of our results are aligned with previously reported trials about the predictive value of LVEF [26,27], which could be due to the different types of participants included. A clinical trial conducted in Israel with 9000 AMI participants reported the highest one-year mortality rate in the group with severe LVEF < 30%. In this group of patients, underlying cardiac clinical features are the main risk factors for mortality, whereas in patients with preserved LVEF ≥ 50%, comorbidities are often related to one-year poor prognosis [28]. Another trial reported a higher frequency of MACE in patients with a moderately reduced ejection fraction (HFmEF) (LVEF 40–49%) compared to those with HFrEF (LVEF < 40%) [29]. In fact, patients without LVEF improvement after myocardial revascularization are at a higher risk of MACE including death [30].

Another study comparing patients with and without coronary artery disease (stable and unstable) reported higher MACE (re-MI, worsening heart failure, recurrent angina) in patients with ACS and Gal-3 levels above the median level. The Gal-3 was an independent predictor for cumulative MACE (increase in Gal-3 by 1 ng/mL led to 6% higher rate of MACE incidence) [17]. Kokturk S and colleagues evaluated 143 patients with ST-elevation myocardial infarction who underwent primary percutaneous intervention and followed up on them for 2 years. High Gal-3 levels were associated with short-term and long-term risk of adverse events (cardiovascular, heart failure and re-hospitalization) but was not an independent predictor of 2-year cardiovascular mortality [31].

All of the above-mentioned studies corroborate our findings; however, the vast majority of these measured the Gal-3 plasma concentration in the peripheral vein, followed up on patients for one year and included varied adverse outcomes as part of MACE. Our previously reported study showed that the Gal-3 determined on the 30th day of AMI in the cubital vein has the highest prognostic value for adverse myocardial remodeling six months later [13]. Grandin EW and colleagues have shown that patients with elevated Gal-3 and BNP levels were at the highest odds of developing HF, suggesting a potential incremental value of Gal-3 for assessment of HF risk after ACS (pilot experience from PROVE IT-TIMI 22) [32]. An association between elevated Gal-3 and adverse post-myocardial infarction remodeling at six months was also found by Perea RJ and colleagues [33]. In a study with 217 patients with AMI, peripheral venous blood samples for Gal-3 plasma concentrations were obtained within 24 h after admission. Gal-3 plasma concentration was an independent predictor of post-MI new onset atrial fibrillation [34]. Erdogan O and colleagues found Gal-3 to be a new biomarker that predicts ventricular arrhythmia in patients with ischemic dilated cardiomyopathy. This study included 51 patients: 19 healthy controls and 32 patients with ischemic dilated cardiomyopathy and previously implanted VVI-ICD. Gal-3 concentrations were higher in the group of patients with arrhythmias requiring ICD therapies vs. those without (*p* = 0.02) and in the group of patients with previously verified arrhythmia storm than in patients without shocks (*p* = 0.05). ROC curve analysis identified patients with a risk of ventricular arrhythmia that required therapies with 84% sensitivity and 75% specificity for Gal-3 levels >7 ng/mL [35]. Takemoto Y and colleagues examined intracardiac serum Gal-3 levels in patients with paroxysmal and persistent AF. Gal-3 levels were higher in patients with persistent AF. In a sheep model of tachypacing-induced AF, they tested the effects of Gal-3 inhibition during AF progression and found that Gal-3 was higher in animals with persistent AF, and the Gal-3 inhibitor GM-CT-01 decreased overall AF burden [36]. 

The novelty brought by this study is reflected in the determination of the Gal-3 plasma concentration from central or peripheral and arterial or venous blood, which, in fact, showed that the aortic root might have the best prognostic value. The sensitivity and specificity based on the Gal-3 plasma concentration from the aortic root in predicting the likelihood of MACE are higher than in other locations and in other studies. A single Gal-3 value from the aortic root during coronary angiography provides strong prognostic value for both MACE and death at six months follow-up. This result also indicates trans-myocardial gradient and possible therapeutic use for Gal-3 inhibitors in high-risk AMI patients with high Gal-3 concentrations in the first 24 h of chest pain. This could be an aim for future investigations.

MACE remains the major cause of mortality and morbidity in patients with AMI. It has no concrete definition. According to our and previously reported studies, Gal-3 has an important role in the post-AMI period in different adverse events. Determining high-risk patients for adverse post-AMI events through simple Gal-3 determination during coronary angiography could be a step forward in the prognosis of AMI patients and prevention in the post-AMI period. Aims for future investigations could be the prognostic power of scoring systems with Gal-3 concentration incorporated into them. Different pathophysiological processes cause an increase in the Gal-3 concentrations at different times and in different locations. Determining its role in positive and negative remodeling, heart rhythm disorders and development of MACE including mortality could be a therapeutic target in this group of patients. 

The main limitation of our study is the small number of participants. The uniqueness of this study is that we measured the Gal-3 plasma concentration in four different blood locations, across the trans-myocardial gradient, during coronary angiography or PCI. We showed that the cubital vein is not the most promising sample type for determining Gal-3’s prognostic value for MACE or death, particularly in a multivariate model. Aortic root Gal-3 appears to show the best compromise between the sensitivity and specificity for the prognosis of MACE or death.

## 5. Conclusions

The Gal-3 plasma concentrations in arterial and venous blood and central and peripheral blood on day one of AMI are increased in patients who develop MACE at six months follow-up. However, the most valuable prognostic biomarker is the Gal-3 plasma concentration collected from the aortic root on day one, which is independently associated with an increased risk of MACE or death at six months post-AMI.

## Figures and Tables

**Table 1:** Galectin-3 (Gal-3) concentrations and baseline characteristics of this study’s patients stratified by major adverse cardiovascular events (MACEs) including death.

	All Patients *N* = 59	No MACE or Death *N* = 39	MACE or Death *N* = 20	*p*-Value
Galectin-3 at site:				
Aortic root (Q1, Q3), ng/mL	9.2 (6.8–12.1)	8.2 (5.9–9.9)	13.9 (10.8–16.2)	<0.001
Femoral/radial artery ± SD, ng/mL	9.72 ± 3.62	8.53 ± 3.46	11.55 ± 3.15	0.005
Coronary sinus ± SD, ng/mL	9.74 ± 3.76	8.43 ± 2.57	12.56 ± 4.40	0.001
Cubital vein ± SD, ng/mL	9.31 ± 3.35	8.05 ± 2.21	12.06 ± 3.80	<0.001
Age ± SD, years	65 ± 9	63 ± 8	69 ± 10	0.013
Gender (male), *n* (%)	42 (71)	32 (78.0%)	9 (50.0%)	0.063
BMI ± SD, kg/m^2^	27.8 ± 3.52	27.8 ± 3.4	27.8 ± 3.9	0.960
Smoking, *n* (%)	18 (31)	14 (34)	4 (22)	0.540
Diabetes mellitus, *n* (%)	20 (34)	14 (70)	6 (30)	0.999
CVI, *n* (%)	3 (5)	2 (5)	1 (6)	0.999
Hypertension, *n* (%)	38 (64)	24 (58)	14 (78)	0.238
Hyperlipoproteinemia, *n* (%)	17 (29)	11 (27)	6 (33)	0.756
STEMI, *n* (%)	38 (64)	23 (56)	15 (83)	0.044
Anterior MI, *n* (%)	26 (44)	16 (41)	10 (50)	0.239
Inferior MI, *n* (%)	33 (56)	23 (59)	10 (50)	0.969
NSTEMI, *n* (%)	21 (36)	16 (41)	5 (25)	0.044
Time from pain onset (Q1, Q3), hours	10.0 (4.0–18.0)	9.5 (4.0–20.0)	8.5 (3.5–15.5)	0.882
AV block, *n* (%)	3 (5)	0 (0)	3 (17)	0.025
VT/VF, *n* (%)	7 (12)	5 (12)	2 (11)	0.999
AF, *n* (%)	5 (8)	2 (5)	3 (17)	0.160
Systolic BP ± SD, mmHg	130 ± 28	137 ± 27	115 ± 26	0.005
Diastolic BP ± SD, mmHg	75 ± 17	79 ± 17	68 ± 15	0.023
HR ± SD, bpm	74 ± 14	76 ± 11	70 ± 21	0.272
Urea (Q1, Q3), mmol/L	6.3 (4.9–8.5)	5.8 (4.6–6.9)	9.6 (7.4–13.3)	0.003
Creatinine (Q1, Q3), μmol/L	87.0 (78.0–100.0)	86.0 (77.0–93.8)	115.5 (79.8–167.8)	0.044
Creatinine clearance ± SD, ml/min	83.41 ± 27.17	91.79 ± 21.29	64.32 ± 29.95	<0.001
Cholesterol ± SD, mmol/L	5.68 ± 1.32	5.88 ± 1.19	5.22 ± 1.51	0.081
HDL ± SD, mmol/L	1.13 ± 0.26	1.12 ± 0.21	1.15 ± 0.35	0.742
LDL ± SD, mmol/L	3.66 ± 1.15	3.86 ± 1.02	3.19 ± 1.33	0.042
Triglycerides (Q1, Q3), mmol/L	1.7 (1.1–2.3)	1.7 (1.2–2.5)	1.3 (1.0–2.0)	0.303
CK-MB (Q1, Q3), U/L	24.0 (15.0–52.0)	24.0 (16.2–54.0)	25.0 (12.2–35.8)	0.987
CRP (Q1, Q3), mg/L	5.0 (1.5–10.6)	4.2 (1.2–10.1)	5.2 (2.7–33.0)	0.278
Troponin T (Q1, Q3), ng/ml	1.2 (0.2–7.3)	1.1 (0.3–5.8)	2.6 (0.1–5.2)	0.593
Pro-BNP (Q1, Q3), pg/ml	459.5 (239.7–2182.7)	372.5 (180.2–1979.5)	2250.0 (275.2–6492.8)	0.004
Glycaemia (Q1, Q3), mmol/L	6.2 (5.3–8.4)	6.0 (5.3–7.0)	8.2 (5.0–12.6)	0.040
Potassium (Q1, Q3), mmol/L	4.3 (4.0–4.6)	4.2 (3.9–4.7)	4.5 (4.1–4.7)	0.281
Sodium (Q1, Q3), mmol/L	139.0 (137.0–141.0)	139.0 (137.2–141.0)	139.5 (136.0–141.2)	0.680
RBC ± SD, ×10^12^/L	4.61 ± 0.56	4.73 ± 0.51	4.30 ± 0.58	0.007
Hemoglobin ± SD, g/L	137.43 ± 18.99	144.44 ± 14.62	120.53 ± 17.91	<0.001
Leukocyte count (Q1, Q3), ×10^9^/L	9.6 (8.6–12.0)	9.4 (8.4–11.6)	10.1 (7.4–12.9)	0.657
Platelet count ± SD, ×10^9^/L	238.03 ± 65.60	232.73 ± 55.37	250.822 ± 80.62	0.406
LVEF ± SD, %	52 ± 5	53 ± 5	50 ± 6	0.157
EDV ± SD, mm	77.62 ± 22.65	76.82 ± 21.73	79.53 ± 25.33	0.683
ESV ± SD, mm	38.25 ± 13.38	38.8 (10.2–45.8)	38.0 (31.5–45.8)	0.392
E/A ratio (Q1, Q3)	0.7 (0.6–0.8)	0.7 (0.6–0.8)	0.7 (0.6–1.0)	0.925
E/E’ ratio (Q1, Q3)	7.9 (6.6–10.0)	7.8 (6.5–9.7)	8.0 (6.6–12.2)	0.263
LA ± SD, mm	37.72 ± 4.93	38.20 ± 4.95	36.59 ± 4.84	0.262
WMA of anterolateral wall, *n* (%)	29 (49)	23 (59)	6 (30)	0.107
WMA of inferolateral wall, *n* (%)	22 (37)	13 (33)	9 (45)	0.181
WMSI	1.40 ± 0.24	1.56 ± 0.29	1.33 ± 0.18	0.001
Number of coronary lesions ± SD	1.67 ± 0.87	1.68 ± 0.82	1.65 ± 1.00	0.887
One-vessel CAD, *n* (%)	11 (19)	8 (21)	3 (15)	0.869
Two-vessel CAD, *n* (%)	20 (34)	15 (38)	5 (25)	0.601
three-vessel CAD, *n* (%)	28 (47)	16 (41)	12 (60)	0.029
Furosemide, *n* (%)	18 (31)	9 (22)	9 (53)	0.030
Spironolactone, *n* (%)	10 (17)	5 (12)	5 (29)	0.139
ACE inhibitors, *n* (%)	42 (71)	32 (78)	10 (59)	0.197
Beta-blockers, *n* (%)	43 (73)	33 (80)	10 (59)	0.107
Calcium channel antagonists, *n* (%)	7 (12)	3 (7)	4 (23)	0.178
Amiodarone, *n* (%)	13 (22)	8 (19)	5 (29)	0.494
DAPT, *n* (%)	53 (90)	39 (95)	14 (82)	0.144
Ticagrelor, *n* (%)	33 (56)	26 (63)	7 (41)	0.151
Trimetazidine, *n* (%)	25 (42)	18 (44)	7 (41)	0.999
Statins, *n* (%)	54 (92)	39 (95)	15 (88)	0.573
UFH/LMWH, *n* (%)	55 (93)	40 (98)	15 (94)	0.999

Data are presented as mean ± SD or median with interquartile range (IQR) or numbers (%). BMI—body mass index; CVI—cerebrovascular insult; STEMI—ST-elevation myocardial infarction; NSTEMI—non-ST-elevation myocardial infarction; AV—atrio-ventricular; VT/VF—ventricular tachycardia/ventricular fibrillation; AF—atrial fibrillation; BP—blood pressure; HR—heart rate; CRP—C reactive protein; Pro-BNP—pro-brain natriuretic peptide; RBC—red blood cell; CAD—coronary artery disease; ACE inhibitor—angiotensin-converting enzyme inhibitor; DAPT—dual antiplatelet therapy; UFH/LMWH—unfractionated heparin/low-molecular-weight heparin; WMA—wall motion abnormalities; WMSI—wall motion score index.

**Table 2:** Major adverse cardiovascular events (MACEs) at 6 months after acute myocardial infarction (AMI).

Event	*n* (%)
MACE, *n* (%)	20 (100)
Death, *n* (%)	5 (25)
Re-AMI, *n* (%)	1 (5)
Cerebrovascular insult, *n* (%)	4 (20)
Re-hospitalization due to heart failure, *n* (%)	5 (25)
Re-hospitalization due to malignant arrhythmias, *n* (%)	5 (25)

MACE—major adverse cardiovascular event; AMI—acute myocardial infarction.

**Table 3:** Multivariate logistic regression for all significant univariate variables (*p* < 0.1) predicting major adverse cardiovascular events (MACEs) or death at six months follow-up.

Univariate Analysis	OR (95%CI)	*p*-Value	R^2^	HL Test *p*-Value
Galectin-3 at site:				
Aortic root	1.277 (1.076–1.517)	0.005	0.255	0.581
Femoral/radial artery	1.309 (1.068–1.604)	0.009	0.224	0.556
Coronary sinus	1.422 (1.155–1.750)	0.001	0.342	0.48695
Cubital vein	1.566 (1.225–2.000)	<0.001	0.405	0.454
Age (years)	1.089 (1.014–1.169)	0.018	0.145	0.289
Gender (male)	0.281 (0.086–0.918)	0.036	0.103	0.301
STEMI	3.913 (0.980–15.625)	0.053	0.101	0.300
NSTEMI	0.256 (0.064–1.020)	0.053	0.101	0.300
Three-vessel CAD, *n* (%)	3.375 (1.110–12.669)	0.033	0.115	0.256
Systolic BP (mmHg)	0.968 (0.945–0.992)	0.009	0.186	0.081
Diastolic BP (mmHg)	0.958 (0.922–0.996)	0.030	0.126	0.553
Urea (mmol/L)	1.546 (1.167–2.048)	0.002	0.296	0.616
Creatinine (μmol/L)	1.034 (1.008–1.061)	0.010	0.264	0.004
Creatinine clearance (ml/min)	0.954 (0.927–0.982)	0.001	0.305	0.220
Cholesterol (mmol/L)	0.677 (0.432–1.062)	0.090	0.074	0.464
LDL (mmol/L)	0.591 (0.349–1.000)	0.050	0.100	0.672
Pro-BNP (pg/mL)	1.000 (1.000–1.001)	0.022	0.176	0.549
Glycaemia (mmol/L)	1.270 (1.043–1.547)	0.017	0.172	0.109
RBC count (×10^12^/L)	0.227 (0.070–0.739)	0.014	0.167	0.913
Hemoglobin (g/L)	0.914 (0.869–0.961)	<0.001	0.439	0.382
WMSI	87.533 (4.311–1777.291)	0.004	0.254	0.791
Furosemide	4.000 (1.198–13.357)	0.024	0.122	0.516
**Multivariate Analysis**	**OR** (**95%CI**)	***p*** **-Value**	**R2**	**HL Test *p*-Value**
Gal-3 level at aortic root ^a^	1.228 (1.011–1.491)	0.038	0.621	0.440
Hemoglobin (g/L)	0.821 (0.699–0.965)	0.017	0.621	0.440
Gal-3 level at femoral/radial artery ^a^	3.438 (1.275–9.265)	0.015	0.846	0.943
Hemoglobin (g/L)	0.860 (0.765–0.966)	0.011	0.846	0.943
Creatinine clearance (ml/min)	0.908 (0.829–0.994)	0.036	0.846	0.943
Gal-3 level at coronary sinus ^a^	1.044 (0.663–1.644)	0.851	0.519	0.858
Hemoglobin (g/L)	0.927 (0.874–0.984)	0.012	0.519	0.858
Gal-3 level at cubital vein ^a^	1.163 (0.694–1.948)	0.566	0.519	0.860
Hemoglobin (g/L)	0.927 (0.874–0.984)	0.012	0.519	0.860
Urea (mmol/L)	1.521 (1.039–2.226)	0.031	0.519	0.860

Dependent variable: major adverse cardiovascular events (MACEs) or death at six months follow-up. Multivariate logistic regression analyses were adjusted for all variables with *p* ≤ 0.1 in univariate analysis. ^a^ Only variable in the model; CI—confidence interval; OR—odd ratio; R^2^—Nagelkerke R square; HL—Hosmer and Lemeshow test; BP—blood pressure; Gal-3—galectin-3; LDL—low-density lipoprotein; pro-BNP—pro-brain natriuretic peptide; RBC—red blood cell; STEMI—ST-elevation myocardial infarction; NSTEMI—non-ST-elevation myocardial infarction; WMSI—wall motion score index.

**Table 4:** The ability of galectin-3 (Gal-3) on day 1 to discriminate patients likely to experience major adverse cardiovascular events (MACEs) or death within 6 months after acute myocardial infarction (AMI).

Gal-3 at Site	AUC (95% CI)	SE	*p*-Value	Cut-Off (ng/mL)	Sn (%)	Sp (%)	PPV (%)	NPV (%)
Aortic root	0.858 (0.744–0.973)	0.058	<0.001	10.86	80%	87%	76%	89%
Femoral artery	0.742 (0.596–0.888)	0.074	0.006	10.18	70%	77%	61%	83%

AUC—area under curve; SE—standard error; CI—confidence interval; Sn—sensitivity; Sp—specificity; PPV—positive predictive value; NPV—negative predictive value.

## Data Availability

All data and supporting materials have been provided with the published article.
